# Selective Influence of Hemp Fiber Ingestion on Post-Exercise Gut Permeability: A Metabolomics-Based Analysis

**DOI:** 10.3390/nu17081384

**Published:** 2025-04-19

**Authors:** David C. Nieman, Camila A. Sakaguchi, James C. Williams, Wimal Pathmasiri, Blake R. Rushing, Susan McRitchie, Susan J. Sumner

**Affiliations:** 1 Human Performance Laboratory, Appalachian State University, North Carolina Research Campus (NCRC), Kannapolis, NC 28081, USA; olsonca1@appstate.edu (C.A.S.); williamsjc12@appstate.edu (J.C.W.); 2 Department of Nutrition, University of North Carolina at Chapel Hill, Chapel Hill, NC 27599, USA; wimal_pathmasiri@unc.edu (W.P.); blake_rushing@unc.edu (B.R.R.); susan_sumner@unc.edu (S.J.S.); 3 Nutrition Research Institute, University of North Carolina at Chapel Hill, North Carolina Research Campus (NCRC), Kannapolis, NC 28081, USA; susan_mcritchie@unc.edu

**Keywords:** hemp fiber, exercise, gut permeability, metabolomics

## Abstract

**Objectives:** This study investigated the effects of 2-week ingestion of hemp fiber (high and low doses) versus placebo bars on gut permeability and plasma metabolite shifts during recovery from 2.25 h intensive cycling. Hemp hull powder is a rich source of two bioactive compounds, N-trans-caffeoyl tyramine (NCT) and N-trans-feruloyl tyramine (NFT), with potential gut health benefits. **Methods:** The study participants included 23 male and female cyclists. A three-arm randomized, placebo-controlled, double-blind, crossover design was used with two 2-week supplementation periods and 2-week washout periods. Supplement bars provided 20, 5, or 0 g/d of hemp hull powder. Participants engaged in an intensive 2.25 h cycling bout at the end of each of the three supplementation periods. Five blood samples were collected before and after supplementation (overnight fasted state), and at 0 h-, 1.5 h-, and 3 h-post-exercise. Five-hour urine samples were collected pre-supplementation and post-2.25 h cycling after ingesting a sugar solution containing 5 g of lactulose, 100 mg of ^13^C mannitol, and 1.9 g of mannitol in 450 mL of water. An increase in the post-exercise lactulose/^13^C mannitol ratio (L:^13^CM) was used as the primary indicator of altered gut permeability. Other outcome measures included muscle damage biomarkers (serum creatine kinase, myoglobin), serum cortisol, complete blood cell counts, and shifts in plasma metabolites using untargeted metabolomics. **Results:** No trial differences were found for L:^13^CM, cortisol, blood cell counts, and muscle damage biomarkers. Orthogonal partial least-squares discriminant analysis (OPLSDA) showed distinct trial differences when comparing high- and low-dose hemp fiber compared to placebo supplementation (R2Y = 0.987 and 0.995, respectively). Variable Importance in Projection (VIP) scores identified several relevant metabolites, including 3-hydroxy-4-methoxybenzoic acid (VIP = 1.9), serotonin (VIP = 1.5), 5-hydroxytryptophan (VIP = 1.4), and 4-methoxycinnamic acid (VIP = 1.4). Mummichog analysis showed significant effects of hemp fiber intake on multiple metabolic pathways, including alpha-linolenic acid, porphyrin, sphingolipid, arginine and proline, tryptophan, and primary bile acid metabolism. **Conclusions:** Hemp fiber intake during a 2-week supplementation period did not have a significant effect on post-exercise gut permeability in cyclists (2.25 h cycling bout) using urine sugar data. On the contrary, untargeted metabolomics showed that the combination of consuming nutrient-rich hemp fiber bars and exercising for 135 min increased levels of beneficial metabolites, including those derived from the gut in healthy cyclists.

## 1. Introduction

Long-distance runners and cyclists have a high incidence of gastrointestinal-related symptoms, and this may be related in part to transient, exercise-induced changes in gastrointestinal integrity and function [1,2]. Bouts of strenuous and intensive cycling and running may alter intestinal permeability, but this varies widely between individuals. Underlying mechanisms for exercise-induced changes in intestinal permeability include reductions in splanchnic blood flow, an increase in sympathetic system activation, hyperthermia, changes in intestinal transporter activity, and selective changes in the tight junctions between intestinal epithelial cells [2]. Nutrition-based strategies may influence exercise-induced changes in gut permeability, including beverage ingestion, to maintain hydration status, ingestion of easily digested carbohydrate-rich foods pre- and post-exercise, and the use of bovine colostrum, glutamine, and probiotic supplements [3,4,5].

Emerging evidence supports that moderate and intensive exercise increases the translocation of gut-derived phenolics from the intestinal tract to the circulatory system following the ingestion of flavonoid-rich supplements and foods [6,7,8]. This may be due, in part, to exercise-induced increases in gut permeability, but other potential underlying mechanisms include changes in gastrointestinal motility and gut transporter function, as well as flavonoid-induced changes in the gut microbial composition [7].

Dietary fibers include nutrients and bioactive compounds that may add to their health benefits. Hemp hull fiber derived from the outer seed coat of hemp is rich in bioactive and insoluble fibers [9,10]. The hull from hemp seeds contains 30–40% fiber, phenolic compounds such as flavones, flavonols, and terpenes, and hydroxycinnamic acid amides with potential gut health benefits, including N-trans-caffeoyl tyramine (NCT) and N-trans-feruloyl tyramine (NFT) [9,10,11]. Few human trials with hemp fiber supplements have been conducted [10]. Cell culture and animal studies indicate some positive influences of NCT and NFT on gut barrier function and gut microbiome composition [9,10,11,12]. Hepatocyte nuclear factor 4α (HNF4α) is a signaling compound and nuclear transcription factor with multiple functions, including the regulation of intestinal permeability, gut barrier function, mucin production, and expression of tight junction proteins. NCT and NFT are agonists of HNF4α [12].

The hypothesis for this investigation was that ingestion of a hemp fiber bar containing NCT and NFT would influence exercise-induced alterations in gut permeability and shifts in related plasma metabolites. This study examined the efficacy of 2-week ingestion of a hemp fiber bar (high and low doses) in altering exercise-induced gut permeability using a randomized crossover design. Exercise-induced alterations in gut permeability were measured using changes in the urine lactulose-to-^13^C mannitol (L:^13^CM) ratio, and plasma metabolites via untargeted metabolomics.

## 2. Materials and Methods

### 2.1. Study Participants

Healthy male and female cyclists were invited to participate in this study if they met the inclusion criteria, including 18 to 65 years of age, capable of cycling 2.25 h in a laboratory setting at 70% maximal oxygen consumption rate (VO_2max_), and a willingness during the 10-week study period to avoid supplements, herbs, and medications with a potential to influence post-exercise metabolic recovery. To be included in the study, study participants had to report that they did not have a gastrointestinal disease (irritable bowel syndrome, chronic nausea, vomiting, diarrhea, Crohn’s disease, Celiac disease, diverticulosis). Participants agreed to taper exercise training and ingest a moderate-carbohydrate diet using a food list restricting high-fat foods and visible fats, and high amounts of black pepper, red pepper, garlic, curry, and other strong spices during the 3-day period prior to each cycling bout.

**Figure 1:**
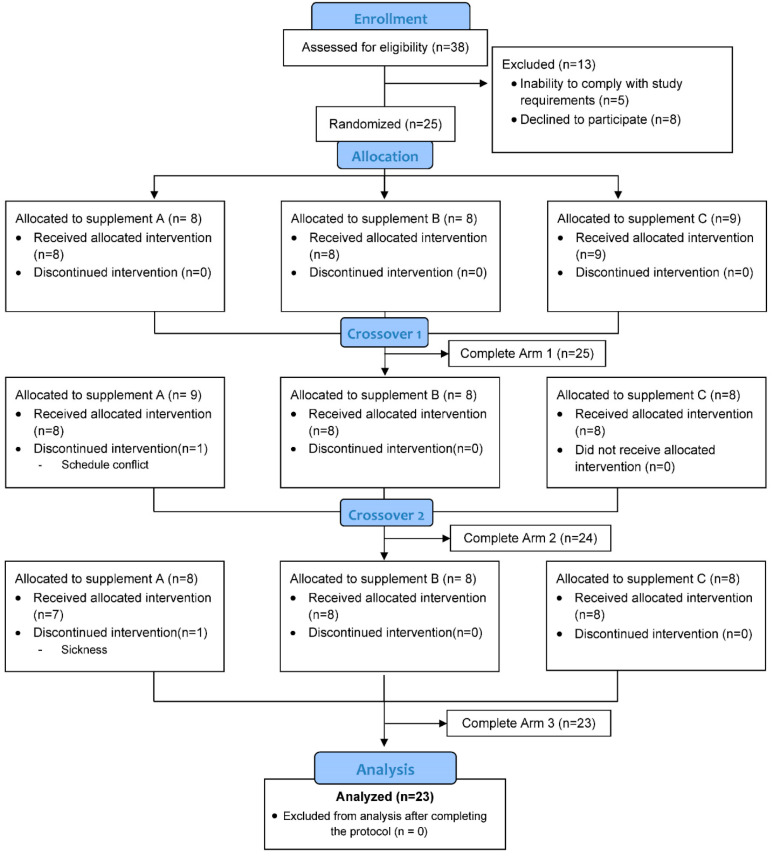
Study participant flow diagram. Thirty-eight participants were assessed for eligibility, and 25 were entered into the study, with 23 completing all aspects of the protocol.

Thirty-eight participants were assessed for eligibility and 25 were entered into the study, with 23 completing all aspects of the protocol (Figure 1). Participants voluntarily signed the informed consent, and procedures were approved by the university’s Institutional Review Board. Trial Registration: ClinicalTrials.gov, U.S. National Institutes of Health, identifier: NCT06204666, approved on 11 January 2024.

### 2.2. Study Design

**Figure 2:**
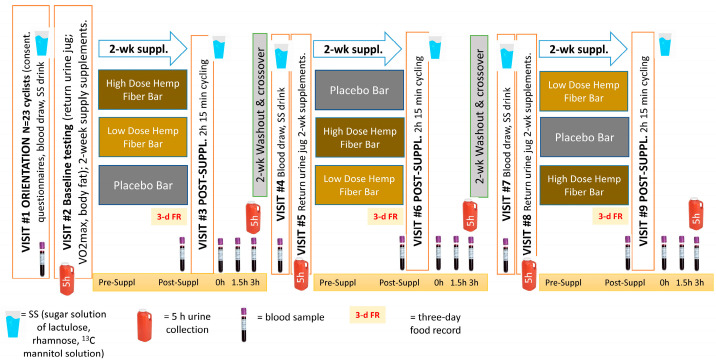
Study design. This study employed a randomized, placebo-controlled, double-blind, crossover design with three 2-week supplementation periods and 2-week washout periods.

This study employed a randomized, placebo-controlled, double-blind, crossover design with three 2-week supplementation periods and 2-week washout periods (Figure 2). Thus, 23 subjects completed each of the 3 trials and functioned as their own controls. Study procedures were conducted at the Appalachian State University Human Performance Laboratory (HPL), North Carolina Research Campus, Kannapolis, NC, USA.

Study participants were block randomized to three trials (high- or low-dose hemp fiber bars or the placebo), and supplements were administered using double-blinded, placebo-controlled procedures. The supplement bars were coded by the sponsor, with the double-blind code held until after all study samples had been analyzed. Subjects came to the lab for orientation/baseline testing, pre- and post-supplementation blood sample collections (2-week supplementation with high- and low-dose hemp fiber bar supplements or placebo), and three 2.25 h cycling sessions (thus, nine total lab visits).

During the first lab visit, study participants reported to the lab in an overnight fasted state, voluntarily signed the consent form, and completed questionnaires, including a 1–10 rating of delayed onset of muscle soreness (DOMS) [13], profile of moods state (POMS) [14], and demographic, health, and training histories. An abbreviated 40-item version of POMS was used, and participants rated moods using the right-now approach [14]. Responses were based on a five-point scale anchored by not at all (score of 0) and extremely (score of 4). Scores for the seven subscales were calculated by summing the numerical ratings for items that contributed to each subscale, with the total mood disturbance (TMD) calculated by summing the totals for the negative subscales (tension, depression, anger, fatigue, confusion) and then subtracting the total for the positive subscales (vigor, esteem-related affect) and adding 100 to eliminate negative scores.

A blood sample was collected during the first lab visit, and participants then ingested a nonabsorbable sugar solution (SS) containing 5 g of lactulose (Sigma Aldrich, St. Louis, MO, USA), 100 mg of ^13^C mannitol (Cambridge Isotope Laboratories, Tewksbury, MA, USA), and 1.9 g of ^12^C mannitol (Sigma Aldrich) in 450 mL of water. An increase in the post-exercise lactulose/^13^C mannitol ratio (L:^13^CM) was used as the primary indicator of increased gut permeability [15]. All urine excreted from 0–5 h post-SS ingestion was collected in a urine collection container. Participants were urged to drink water after the 1st hour of SS ingestion to ensure adequate urine output. The Boost beverage was consumed at 7 kcal/kg 1.5 h post-SS ingestion (to simulate what occurred in this study post-exercise). The urine collection container was placed in the refrigerator until they returned to the lab the next day. The total urine volume was measured, and four 50 mL aliquots were frozen in a minus 80 °C freezer until analysis.

During the second lab visit the next day, participants returned the urine collection container. Height and body weight were assessed, with body composition measured using the BodPod system (Cosmed, Rome, Italy). Study participants were tested for maximal aerobic capacity (VO_2max_) during a graded, cycling test with the Lode cycle ergometer (Lode B.V., Groningen, The Netherlands) and the Cosmed CPET metabolic cart (Cosmed, Rome, Italy).

A 2-week supply of high-dose or low-dose hemp fiber bars or placebo bars was provided to the participants (after block randomization). Subjects consumed two bars per day, one with the first meal in the morning and the second bar with the last meal of the day. Hemp hulls are the hard, outer hemp seed shell, which are left over after the dehulling process to extract hemp hearts. The study sponsor (Brightseed, San Francisco, CA, USA) transformed the hemp hulls into a dietary fiber ingredient with a high concentration of NCT and NFT (Brightseed Bio 01^™^) (https://www.brightseedbio.com/bioactives/bio-gut-fiber (accessed on 1 August 2024)). Certificates of analysis indicated no cannabinoid content or micro-biological contamination. The ingredients in the bars are common and at a food-grade level (Table 1). The three types of bars contained comparable energy and macronutrient content.

To facilitate compliance with the supplementation protocol, study participants were contacted via email on a regular basis and returned the bar wrappers at the end of the supplementation period.

During the 3-day period prior to the 2.25 h cycling sessions, participants tapered exercise training and ingested a moderate-carbohydrate diet using a food list restricting high-fat foods, visible fats, and caffeine. Participants recorded all food and beverage intake in a 3-day food record with macro- and micro-nutrient intake calculated using the Food Processor dietary analysis software system (Version 11.11, ESHA Research, Salem, OR, USA).

Study participants reported to the Human Performance Lab in an overnight fasted state and provided a blood sample, ingested the supplement (one high- or low-dose hemp fiber bar or placebo bar), and then cycled 2.25 h at high intensity (~70% VO_2max_) while ingesting water alone (3 mL/kg every 15 min). Immediately following the cycling bout, subjects ingested the SS. Blood samples were collected at 0 h, 1.5 h, and 3.0 h post-exercise. All urine excreted for 5 h after SS ingestion was collected. The testing protocol during the lab sessions with the 2.25 h cycling sessions was organized as follows:

- 7:00 a.m.: Participants turned in the 3 d food record. A 30 mL blood sample was collected. Participants provided DOMS and POMS ratings and completed a 2-week retrospective symptom survey (with ratings of gastrointestinal symptoms, mental health, respiratory illness, sleep quality, pain symptoms, and overall well-being).
- 7:10 a.m.: Participants ingested one supplement bar with one cup of water.
- 7:30 a.m.: After a warm-up, participants cycled for 2.25 h at approximately 70% VO_2max_ on their own bicycles fitted to Saris H3 direct drive smart trainers (Madison, WI, USA) with monitoring by the Zwift online training platform (Long Beach, CA, USA) and the Cosmed CPET metabolic cart (Rome, Italy). Heart rate, cycling speed, cadence, distance, and power were measured and recorded continuously during the 2.25 h bout. Metabolic parameters such as breathing rate, ventilation, and oxygen intake were measured after 15 min and then every 30 min during the cycling session. To ensure performance consistency between trials, performance data from the first trial was used to ensure a similar power and metabolic output during the second and third trials. Participants consumed 3 mL/kg of water every 15 min. No other beverage or food containing energy or nutrients was allowed during the 2.25 h cycling sessions.
- 3 h post-exercise period: Participants ingested 450 mL of SS within the 1st minute of getting off the bicycle, and urine was collected for the next 5 h. Blood samples were collected immediately after completing the cycling session, and then 1.5 h and 3.0 h post-exercise. Participants were allowed to shower and change their clothes. The DOMS and POMS questionnaires were administered each time blood samples were collected. No food or beverage other than water (7 mL/kg) was ingested during the first 1.5 h post-exercise. After the 1.5 h post-exercise blood draw, participants ingested a fortified nutrient beverage (Boost, Nestlé S.A., Vevey, Switzerland). Another blood sample was collected 3 h post-exercise. Afterwards, participants were allowed to stay in the lab to complete the 5 h urine collection or leave the lab and return later in the day to turn in the 5 h urine container.

After the first two cycling sessions, participants completed a 2-week washout period without the supplements, crossed over to the next treatment arm, and then repeated all procedures. Participants maintained their normal diets and exercise routines during the 2-week washout periods.

### 2.3. Sample Analysis

Plasma aliquots were prepared from EDTA blood collection tubes and stored in a −80 °C freezer for metabolomics analysis. The 5 h urine samples were weighed with aliquots prepared and stored in a −80 °C freezer for sugar analysis. Serum creatine kinase, myoglobin, and cortisol (from serum separator tubes), and complete blood counts (CBCs) with a white blood cell differential count (EDTA tubes), were analyzed using Labcorp services (Burlington, NC, USA).

#### 2.3.1. Urine Sugar Analysis

The urine samples were analyzed using a high-performance liquid chromatography (HPLC) method for ^12^C- and ^13^C-mannitol, and lactulose at the Mayo Clinic’s Immunochemical Core Lab (Rochester, MN, USA) [16]. The HPLC-MS/MS System included an API 5000 triple-quadruple mass spectrometer (Applied Biosystems/MDS SCIEX, Foster City, CA, USA/Concord, Vaughan, ON, Canada) coupled with an electrospray ionization source that was operated at 700 °C in the negative ion mode. Urine samples (25 µL) were added to a 96-deep-well plate. Samples, quality controls, and calibrators were diluted 11-fold by the addition of 250 µL of an internal standard consisting of a mixture of ^13^C-mannitol and lactulose. The analytes were separated by normal-phase HPLC and detected on a tandem mass spectrometer (LC-MS/MS) utilizing electrospray ionization, operating in the multiple-reaction monitoring negative mode. The calibration utilized two different six-point standard curves over a concentration range of 0.5–500 µg/mL for mannitol and 0.125–125 µg/mL for lactulose. The ^13^C-mannitol internal standard was used to normalize the mannitol values, and the lactulose internal standard normalized the lactulose values. Sugar peaks were identified and measured using Analyst 1.6 software package (MDS SCIEX, Concord, Vaughan, ON, Canada). The limit of detection, the lowest analyte concentration likely to be reliably distinguished from the limit of blank, was 0.3 mg/mL for ^12^C-mannitol, 0.5 mg/mL for ^13^C-mannitol, and 0.3 mg/mL for lactulose.

#### 2.3.2. Plasma Untargeted Metabolomics Analysis and Statistical Procedures

The untargeted metabolomics analysis procedures have been described in detail elsewhere [17,18,19,20]. Briefly, untargeted metabolomics data of randomized plasma samples (interspersed with 10% blanks, quality-control study pools (QCSP), and NIST SRM 3672 reference material) were acquired in positive mode on a Vanquish UHPLC system coupled with a Q Exactive^™^ HF-X Hybrid Quadrupole-Orbitrap^™^ Mass Spectrometer (UHPLC-HRMS; Thermo Fisher Scientific, San Jose, CA, USA). One subject in the low-dose hemp fiber study trial had the immediate-post-exercise blood sample excluded due to a technical error in acquiring the metabolomics data. Raw data files for all study samples, QCSP, blank, and NIST reference material runs were uploaded to Progenesis QI (Waters Corporation, Milford, MA, USA) for alignment and peak picking. Data were normalized to a reference QCSP sample using the “normalize to all” function in Progenesis QI [21,22]. Peaks detected by UHPLC-HRMS were identified or annotated using ADAP-KDB software (Version 1.8.5, https://adap.cloud/ accessed on 1 August 2024) to in-house reference libraries and public databases [23] (Supplementary Table S1). The evidence basis for metabolite identifications and annotations was denoted using an ontology system as previously described [20]. As is the case with LC-MS-based platforms, isomers may not always be distinguishable. Names provided for each match are based on the names of the reference standards run on the UHPLC-HRMS platform.

The difference in peak intensity for each arm was calculated by subtracting the pre-study intensity from the post-supplementation and post-exercise peak intensities. A linear mixed model accounting for unequally spaced repeated measures with a spatial power law covariance structure was created for each peak using SAS^®^ 9.4 (SAS Institute Inc., 2023, Cary, NC, USA) to determine whether there was a significant effect of hemp fiber (high and low doses) supplementation compared to placebo (Supplementary Table S2). One subject was excluded due to missing data at the immediate post-exercise time point.

The difference in peak intensity for each trial was also modeled using orthogonal partial least-square discriminant analysis (OPLSDA; (SIMCA 18, Sartorius Stedim Data Analytics, AB, Umeå, Sweden)), which is a multivariate method frequently used for analyzing high-dimensional collinear data. The variable importance to projection (VIP) statistic allows for the identification of peaks important to the differentiation of the groups, and peaks with a VIP ≥ 1 were defined as differentiators. The model statistics include R2Y, which is the percentage of variation in the differentiation of the groups explained by the model, and Q2, which is based on a 7-fold cross-validation that provides an assessment of predictive ability of the model.

### 2.4. Additional Statistical Procedures

Data are expressed as mean ± SE. Except where described, datasets were analyzed using the generalized linear model (GLM), repeated measures ANOVA module in SPSS (IBM SPSS Statistics, Version 28.0, IBM Corp, Armonk, NY, USA). The statistical model utilized the within-subjects approach: three (trials) × 5 (time points) repeated measures ANOVA and provided time (i.e., the collective effect of the cycling exercise bout), supplement (i.e., the collective supplement effect), and interaction effects (i.e., whether the data pattern over time differed between trials). If the interaction effect was significant (*p* ≤ 0.05), then post-hoc analyses were conducted using paired t-tests comparing time point contrasts between trials. An alpha level of *p* ≤ 0.0125 was used after Bonferroni correction for four multiple tests.

## 3. Results

As summarized in Figure 1, 23 of 25 study participants who were entered in the study completed the three arms of the study and related requirements. Table 2 summarizes characteristics for the male (*n* = 16) and female (*n* = 7) cyclists. Performance data for each of the three trials are summarized in Table 3. As designed for this study, no trial differences were found for the performance measurements when analyzed by sex or for all participants combined. The pattern of change over time did not differ between the male and female cyclists for the primary outcome of this study (L/M) (supplement × time × sex interaction effect, *p*-value = 0.502) and plasma disaccharides from the untargeted metabolomics analysis (*p* = 0.893). Thus, outcome measures are presented for all study participants combined.

Three-day food records were collected at the end of each 2-week supplementation period and macronutrient intake did not differ significantly between the three arms. Nutrient data from the 3-day food records were averaged, and the mean energy intake was 2184 ± 136 kcal/day (9.14 ± 0.57 MJ/day), and carbohydrate, protein, fat, and alcohol represented 45.7 ± 1.5, 19.5 ± 1.0, 34.1 ± 1.2, and 2.1 ± 0.7%, respectively, of total energy. Total flavonoid intake from the background diet averaged 97.4 ± 24.2 mg/day.

Data from the 2-week retrospective symptoms logs recorded at the end of each supplementation period indicated that gastrointestinal, mental health, respiratory illness, and sleep-quality symptoms did not differ significantly when ingesting the high- and low-dose hemp fiber supplements compared to placebo. Study participants were highly compliant, with over 98.5% of the supplement bars ingested during each of the three trials. A post-study questionnaire at the end of each supplement period revealed that study participants correctly guessed 33% of the time what type of supplement they were ingesting, with 18% and 48% indicating incorrect or “don’t know” responses (Χ^2^ = 9.09, *p* = 0.011). Correct responses were especially noted when participants consumed the high-dose hemp fiber bars (59%).

The neutrophil/lymphocyte ratio, serum cortisol, myoglobin, and creatine kinase concentrations, and DOMS increased post-exercise in the three arms of the study (all-time effects *p* < 0.001). No differences were found for the patterns of change over time for each of these parameters (all interaction effects, *p* > 0.20).

The gut permeability data, including urine L:C^13^M and L:C^12^M ratios, are summarized in Table 4. The pattern of change from the pre-supplementation to post-exercise 5 h urine samples for each of these parameters did not differ significantly between the three supplementation trials. Analysis of three combined plasma disaccharides from the metabolomics dataset (sucrose, lactose, maltose) across all five timepoints showed no increases post-exercise (time effect, *p* = 0.434) or trial differences (interaction effect, *p* = 0.459).

**Figure 3:**
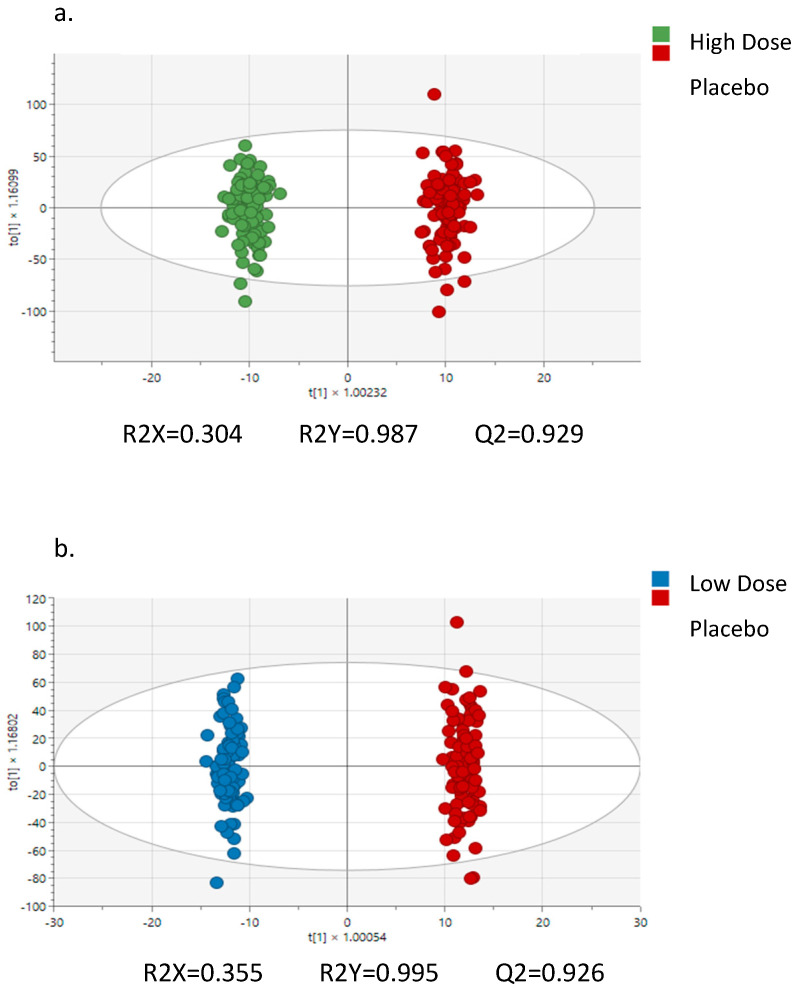
(**a**,**b**) OPLSDA analysis of high− and low−dose hemp study arms relative to placebo. Data represent metabolite changes from pre−supplementation time points across each of the four other time points (post−2 weeks supplementation, immediately post−2.25 h cycling, and 1.5 h and 3 h post-exercise.

The OPLSDA analysis of the untargeted metabolomics data from the plasma samples is depicted in Figure 3a (high-dose hemp fiber versus placebo) and Figure 3b (low-dose hemp fiber versus placebo). This analysis used change data calculated by subtracting pre-supplementation peak metabolite intensities from each of the other four time points (post-2 weeks supplementation, immediately post-exercise, and 1.5 h and 3 h post-exercise. R2Y and Q2 data indicate strong trial separations that were highly reproducible. Table 5 summarizes the high evidence-based metabolites that had variable importance to projection (VIP) scores of 1.4 and higher, FDR *p*-values of <0.05, and Mummichog pathway confirmation as a plausible and related metabolite when comparing the high-dose hemp fiber with the placebo trials. See Supplementary Table S3 for the full list of metabolites important to differentiating the study trials.

**Figure 4:**
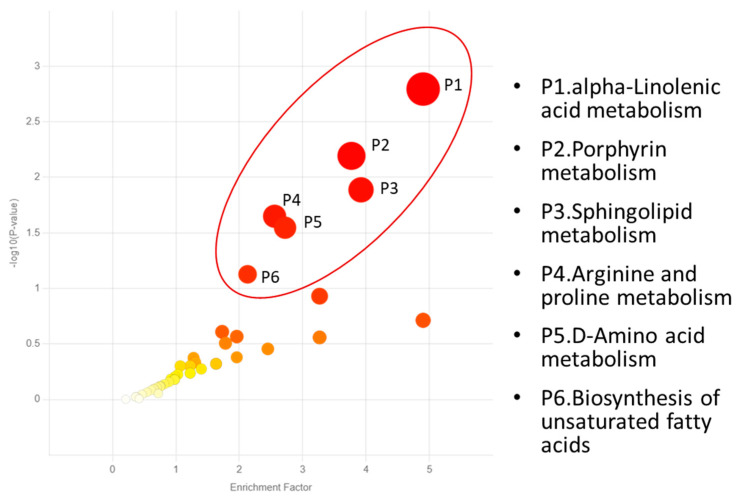
Six metabolic pathways were significantly influenced by hemp fiber supplementation across all time points (interaction effects, *p* < 0.05). The color and size of each circle corresponds to its *p*-value and enrichment factor, respectively. The large red circles indicate the most significant pathways influenced by hemp fiber supplementation.

**Figure 5:**
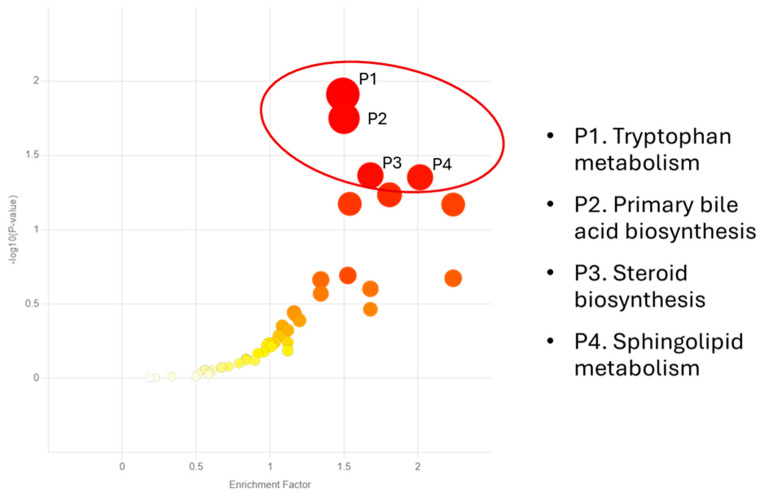
Four metabolic pathways were significantly influenced by hemp fiber supplementation across all time points (supplement main effects, *p* < 0.05). The color and size of each circle corresponds to its p-value and enrichment factor, respectively. The large red circles indicate the most significant pathways influenced by hemp fiber supplementation.

The Mummichog pathway analysis identified six biochemical pathways that were significantly influenced by hemp fiber supplementation (interaction effects, each *p* < 0.05) (Figure 4). Table 6 lists the KEGG metabolites that were significantly changed within each pathway. Additionally, an additional analysis using supplement main effects (*p* < 0.05) established four pathways, including (P1) tryptophan metabolism, (P2) primary bile acid biosynthesis, (P3) steroid biosynthesis, and (P4) sphingolipid metabolism (Figure 5). Key and significant metabolite hits within the tryptophan pathway included tryptophan, serotonin, 5-hydroxy-trypthophan (5-HTP), 5-hydroxyindoleacetic acid, kynurenine, and indoleacetic acid. Numerous metabolites with significant hits within the primary bile acid biosynthesis pathway included 27-hydroxycholesterol, 7α-hydroxycholesterol, chenodeoxycholic acid, cholic acid, and glycocholic acid. Significant metabolite hits within the steroid biosynthesis pathway included vitamin D3, calcidiol, 7-dehydrocholesterol, and calcitriol.

## 4. Discussion

The study hypothesis was based primarily on cell culture and animal study data [9,10,11] and targeted the potential linkage between 2-week ingestion of supplement bars containing 5 and 20 g/d hemp hull fiber enriched with NCT and NFT and alterations in post-exercise gut permeability. The hull from hemp seeds is rich in insoluble dietary fiber, phenolic compounds, and hydroxycinnamic acid amides, including NCT and NFT [9,10,11,12]. NCT and NFT are agonists of HNF4α, which has some regulatory effects on gut barrier integrity and function, as well as lipid metabolism [10,24].

A strong research design was employed for outcome measures, including the analysis of pre-supplementation and post-exercise 5h urine samples for the L:^13^CM ratio [15,16]. Untargeted metabolomics was utilized to measure shifts in metabolites related both to gut permeability and the ingestion of hemp hull fiber bars. No trial differences were found for L:^13^CM, but metabolomics showed distinct trial differences for gut-derived metabolites. Multiple metabolic pathways were enriched with hemp fiber intake, including those linked to alpha-linolenic acid, porphyrin, sphingolipid, arginine and proline, tryptophan, and primary bile acid metabolism. Few randomized human clinical trials have been conducted using untargeted metabolomics to characterize shifts in metabolites linked to increased dietary fiber intake [25,26,27,28]. Dietary fibers are metabolized by gut bacterial species, resulting in a wide array of metabolites that enter the circulation and influence multiple metabolic pathways [27]. The hemp fiber bars added significant amounts of dietary fiber to the diets of our subjects and caused widespread shifts in circulating metabolites from many different pathways. Dietary fiber supplement studies often focus on gut-derived short-chain fatty acids (SCFA). This study used the positive mode for mass spectrometry, and SCFAs were not detected. The SCFA response is dependent on the dose and the type and structure of dietary fibers. Soluble fibers are quickly fermented by the gut microbiota in the colon and increase SCFAs. Conversely, insoluble fibers, as derived from hemp hulls, increase the rate of passage through the large intestine and contribute to fecal bulking but cause less fermentation and SCFA generation [27,28,29,30].

Several gut-derived metabolites from hemp fiber polyphenols and amino acids were detected in the plasma, including high-VIP metabolites such as 3-hydroxy-4-methoxybenzoic acid, a plant metabolite (isovanillic acid) with antibacterial properties [31]. Other gut-derived metabolites included indolelactic acid from the metabolism of tryptophan, which functions as an anti-inflammatory molecule [32], and 4-Methoxycinnamic acid, which is a methyl derivative of ferulic acid that has been found in hemp fibers [33]. This is the first metabolomics-based study of metabolite shifts due to the combined effect of increased hemp fiber intake and vigorous exercise. Previous research conducted by our research group supports the finding of an increased release of gut-derived metabolites due to the combined influence of polyphenol intake and exercise [7].

Exercise-induced changes in gut permeability depend on many factors, including intensity and duration, heat stress, and hydration status [1,2]. This study utilized an intensive 2.25 h cycling bout in a neutral laboratory environment, and measured changes in L:^13^CM and L:^12^CM were modest and variable. One review concluded that vigorous endurance exercise lasting at least 60 min at 70% VO_2max_ was a sufficient stimulus to induce increased intestinal permeability [1]. However, this is not a consistent finding. For example, in one study [34], a single 60 min bout of intensive cycling induced splanchnic hypoperfusion but failed to increase intestinal permeability when using the urine lactulose/L-rhamnose (L/R) ratio. The same study reported a mild and transient increase in plasma L/R [34]. The current study showed no post-exercise increases in plasma disaccharides using data from the untargeted metabolomics analysis. Thus, the modest and inconsistent increases in post-exercise urine L:^13^CM and L:^12^CM left little room for hemp hull fiber enriched with NCT and NFT to exert a countermeasure effect.

The intestinal epithelium is a complex, selectively permeable barrier that facilitates the absorption and secretion of biochemicals needed to support health and normal physiological processes while acting as a barrier against intraluminal bacteria [35]. Pathological conditions can compromise the integrity of the intestinal barrier, allowing pathogens to translocate into the circulation and triggering inflammation [35,36]. Although long-distance cyclists and runners experience a high incidence of gastrointestinal symptoms and regular transient increases in gut permeability, inflammation, and oxidative stress [37], most studies indicate an overall chronic state of enhanced gut health and integrity in athletes [2]. The presence of lipopolysaccharide (LPS) in the circulation is evidence of bacterial translocation from the gut. We previously showed that blood levels of LPS in long-distance runners were linked to regular ibuprofen use, and that LPS did not increase in non-ibuprofen athletes even after completing the arduous 160 km Western States Endurance Run [38].

Keirns et al. [2] advanced the concept that exercise training can be viewed as a hermetic stressor that promotes beneficial changes in gut barrier function similar to the acute exercise-induced increases in inflammatory biomarkers that underly anti-inflammatory adaptations. Thus, acute changes in gut barrier function do not appear to offset long-term positive influences on the gut barrier. Taken together, the entire concept of ingesting nutritional products to mitigate post-exercise increases in gut permeability has questionable merit in healthy athletes.

The gut microbiome plays a crucial role in maintaining a healthy gut barrier by producing metabolites that influence the tight junctions between intestinal epithelial cells [39]. Although data are limited, the combination of increased dietary fiber and polyphenol intake with regular exercise training may improve gut barrier function in athletes by promoting a healthier gut microbiome [40,41,42]. This approach increases the production and release of gut-derived metabolites that help maintain gut barrier integrity and function. A transient increase in gut permeability and transporter activity has been speculated as a possible mechanism by which vigorous exercise accelerates the movement of beneficial gut-derived phenolics from the lower intestine into the blood [6,7,8]. In previous studies, we showed that long-distance endurance running and cycling enhanced the plasma gut-derived phenolic signature [6,7,8]. We also showed that in the resting state, plasma levels of gut-derived metabolites in long-distance runners were 40% higher than in non-runners [7]. Although speculative, the transient post-exercise elevation in circulating gut-derived phenolics may play a role in diminishing inflammation and oxidative stress during recovery from intensive running [6,7,8]. There is increasing evidence that gut-derived phenolics have wide-ranging bioactive effects on multiple enzyme systems, exerting anti-inflammatory, anti-viral, and immune cell signaling influences, with an enhancement of endothelial health and function in the intestine and vasculature [43].

## 5. Conclusions

This study showed that a 2-week intake of hemp hull fiber did not influence modest changes in gut permeability following 2.25 h of vigorous cycling. However, untargeted metabolomics revealed distinct trial differences when comparing metabolite shifts with high- and low-dose hemp fiber compared to placebo supplementation. The combination of consuming nutrient-rich hemp fiber bars and intensive exercise increased levels of beneficial metabolites, including those derived from the gut in healthy cyclists. Multiple metabolic pathways were influenced by the combination of hemp hull fiber intake and vigorous exercise. Taken together, these data indicate that the combination of hemp hull fiber with 2.25 h cycling had a selective effect on gut permeability and a significant influence on lipid-, bile acid-, and amino acid-related metabolic pathways.

## Figures and Tables

**Table 1:** High dose, low dose, and placebo bar ingredients and macronutrient composition.

Ingredient Name	High-Dose Weight (g)	Low-Dose Weight (g)	Placebo Weight (g)
Slurry Binder	27.45	27.45	0
Hemp Hull Powder	10.00	2.50	0
Colorant	0.05	0.10	0.30
Sunflower oil	1.00	1.00	1.00
Plain Rice Crisps	11.50	11.50	11.50
Milk powder blend	0	7.45	9.75
Tapioca and rice flour binder	0	0	27.45
Total weight (g)	50.00	50.00	50.00
Kilocalories	182	192	189
% carbohydrate	84	77	75
% fat	10	16	18
% protein	5	7	7

**Table 2:** Study participant body composition and maximal cardiorespiratory fitness characteristics (*n* = 23) for male (M) (*n* = 16) and female (F) (*n* = 7) cyclists. * Sex contrast, *p*-value ≤ 0.05.

	Sex	Mean ± SE
Age (yrs)	M	45.5 ± 2.2
	F	46.5 ± 4.1
Weight (kg)	M	79.2 ± 2.1 *
	F	61.7 ± 2.9
Height (cm)	M	181 ± 1.4 *
	F	165 ± 0.6
BMI (kg/m^2^)	M	24.2 ± 0.5
	F	22.6 ± 1.1
Body fat (%)	M	19.4 ± 1.5 *
	F	28.0 ± 2.0
V0_2max_ (ml^.^kg^−1^min^−1^)	M	43.5 ± 1.7 *
	F	34.5 ± 3.0
Max watts	M	265 ± 10.9 *
	F	167 ± 19.0
Max heart rate (beats/min)	M	172 ± 2.7
	F	171 ± 5.5
Max ventilation (L/min)	M	128 ± 6.0 *
	F	79.0 ± 7.5
Max respiratory rate (breaths/min)	M	47.1 ± 2.0
	F	40.0 ± 1.5

**Table 3:** Cycling performance outcomes for the three supplement trials (high-dose and low-dose hemp fiber, and placebo) (*n* = 23 male and female cyclists combined) (mean ± SE). No significant trial differences in average 2.25 h cycling performance measurements were found as designed. For both trials, lab temperature averages 24.3 ± 0.2 °C and humidity is 46.5 ± 1.7%.

Performance Measurement Supplement		Mean ± SE
Cycling power (watts, % maximum)	High-dose hemp	138 ± 9.3 (57.2 ± 1.8% max)
	Low-dose hemp	138 ± 8.6 (57.6 ± 1.4% max)
	Placebo	137 ± 8.6 (57.1 ± 1.3% max)
Heart rate (beats/min, % maximum)	High-dose hemp	134 ± 3.4 (78.4 ± 1.5% max)
	Low-dose hemp	133 ± 3.1 (77.5 ± 1.4% max)
	Placebo	133 ± 3.6 (77.5 ± 1.8% max)
Oxygen consumption (VO_2_) (mL·kg^−1^min^−1^, % maximum)	High-dose hemp	30.1 ± 1.1 (73.8 ± 1.5% max)
	Low-dose hemp	29.9 ± 1.2 (73.1 ± 1.5% max)
	Placebo	29.5 ± 1.2 (72.2 ± 1.5% max)
Distance cycled (km)	High-dose hemp	66.1 ± 2.1
	Low-dose hemp	66.2 ± 1.6
	Placebo	65.8 ± 1.8
Speed (km/h)	High-dose hemp	29.0 ± 0.9
	Low-dose hemp	29.3 ± 0.7
	Placebo	28.6 ± 0.8

**Table 4:** Gut permeability data. Urine (5 h timed samples) lactulose to ^13^C and ^12^C mannitol (L:C^13^M) (L:C^12^M) ratios.

	High-Dose Hemp		Low-Dose Hemp		Placebo		Time and Interaction Effects, *p*-Values
	Pre-Suppl.	Post-Exerc.	Pre-Suppl.	Post-Exerc.	Pre-Suppl.	Post-Exerc.	
L:C^13^M	0.518 ± 0.051	0.780 ± 0.230	0.450 ± 0.028	0.613 ± 0.077	0.498 ± 0.039	0.490 ± 0.028	0.108; 0.195
L:C^12^M	0.031 ± 0.003	0.053 ± 0.002	0.027 ± 0.002	0.037 ± 0.004	0.033 ± 0.003	0.031 ± 0.002	0.070; 0.172

**Table 5:** Metabolites matched with a high evidence basis (ontology metabolites level 1 and selected 2a) that had variable importance to projection (VIP) scores of 1.4 and an FDR-corrected *p*-value < 0.05, and Mummichog pathway analysis confirmation as a plausible and related metabolite when comparing the high-dose hemp fiber and placebo supplement trials. See Supplementary Table S3 for the full list of metabolites important to differentiating the study trials.

VIP	Metabolites	Description
2.3	Uridine	A pyrimidine nucleoside involved with many biological processes, including RNA, glycogen, and biomembrane synthesis.
2.0	Linoleic acid	An essential polyunsaturated fatty acid (18:2ω6).
1.9	Uric acid	Chemical created when purines are metabolized. Uric acid is a significant antioxidant in the human body.
1.9	Valyl-Serine	Dipeptide formed from L-valine and L-serine residues. Incomplete breakdown product of protein digestion or protein catabolism.
1.9	O-Cresol	A derivative of phenol and an isomer of p-cresol and m-cresol. Phenol is primarily used to synthesize plastics and related materials.
1.9	3-hydroxy-4-methoxybenzoic acid	A plant metabolite (isovanillic acid) with antibacterial properties.
1.8	Stearidonic acid	A plant-based omega-3 fatty acid (18:3 *n*-3) that increases the levels of long-chain omega-3 PUFAs such as EPA.
1.8	Glycerophosphocholine	A choline derivative involved in multiple brain functions.
1.8	Creatinine	An endogenous product of muscle metabolism.
1.8	2-Aminoheptanoate	An alpha amino acid. May enhance the effect of ketones as fuel to the Krebs cycle in the brain.
1.8	Quinaldic acid	A kynurenine metabolite.
1.7	Phenylacetylglutamine	A novel metabolite derived from gut microbial metabolism of dietary proteins, specifically phenylalanine, which may be linked to risks of adverse cardiovascular events.
1.7	Indolelactic acid	Formed primarily from gut bacterial metabolism of tryptophan. Functions as an anti-inflammatory molecule.
1.5	Serotonin	Made from tryptophan, an essential amino acid. A chemical messenger that affects mood, sleep, and digestion.
1.5	N-Butyrylglycine	An acyl glycine that is a minor metabolite of fatty acids.
1.5	Cytidine	A pyrimidine nucleoside that serves as a precursor for uridine and is involved in RNA synthesis.
1.5	S-Allylcysteine	An organosulfur compound that exhibits antioxidant, anti-inflammatory, and redox modulatory activities
1.5	Adipoyl-L-carnitine	An acylcarnitine.
1.5	12,13-DiHOME	An inflammatory oxylipin.
1.5	N-Acetylmethionine	A derivative of methionine.
1.5	Calcifediol	The precursor for calcitriol, the active form of vitamin D.
1.4	5-Hydroxytryptophan	Metabolite of tryptophan and the immediate precursor of the neurotransmitter serotonin.
1.4	4-Methoxycinnamic acid	A methyl derivative of ferulic acid that has been found in hemp fibers.
1.4	Proline	An amino acid important in protein synthesis, nutrition metabolism, wound healing and immunity, and antioxidative reactions.
1.4	5-Aminolevulinic acid	An amino acid that is the first compound in the porphyrin synthesis pathway leading to heme.
1.4	Procyanidin B1	A flavonoid group of condensed flavan-3-ols that can be found in many plants.

**Table 6:** Metabolites with KEGG identification numbers significantly changed by hemp fiber supplementation (both high and low doses) within each of the six pathways.

Pathway.	Kegg ID and Metabolites
P1. Alpha-linolenic acid metabolism	C06427 Alpha-linolenic acid C16300 Stearidonic acid
P2. Porphyrin metabolism	C00931 Porphobilinogen C00430 5 Aminolevulinic acid C00486 Bilirubin
P3. Sphingolipid metabolism	C06124 Sphingosine 1-phosphate C00836 Sphinganine C00319 Sphingosine
P4. Arginine and proline metabolism	C00555 4-Aminobutyraldehyde C05147 Trans-3-hydroxy-L-proline C00763 D-Proline C01157 4-Hydroxyproline C01165 L-Glutamic gamma-semialdehyde C00077 Ornithine C03564 1-Pyrroline-2-carboxylic acid C00022 Pyruvic acid C03912 1-Pyrroline-5-carboxylic acid
P5. D-Amino acid metabolism	C00819 D-Glutamine C00515 D-Ornithine C00763 D-Proline C03440 cis-4-Hydroxy-D-proline C01110 5-Amino-2-oxopentanoic acid C03564 1-Pyrroline-2-carboxylic acid
P6. Biosynthesis of unsaturated fatty acids	C00712 Oleic acid C01595 Linoleic acid C00219 Arachidonic acid C06426 gamma-Linolenic acid C06428 Eicosapentaenoic acid C06427 alpha-Linolenic acid

## Data Availability

The raw data supporting the conclusions of this article will be made available by the authors without undue reservation. Metabolomics data have been deposited into the National Metabolomics Data Repository (metabolomicsworkbench.org) with the dataset identifier (the final identifier will be released prior to publication). The metabolomics data from this study (datatrack_id:5717 study_id:ST003806) are now temporarily available at: https://dev.metabolomicsworkbench.org:22222/data/DRCCMetadata.php?Mode=Study&StudyID=ST003806&Access=FrcH9986 (accessed on 24 March 2025). The DOI for this project (PR002380) is: http://dx.doi.org/10.21228/M80G13.

## Supplementary Materials

The following supporting information can be downloaded at: https://www.mdpi.com/article/10.3390/nu17081384/s1, Supplementary Table S1: Metabolites identified or annotated using the in-house physical standards library and public databases; Supplementary Table S2: Linear mixed model with a spatial power law covariance structure: fixed effects; Supplementary Table S3: Peaks Important to Differentiating High-/Low-Hemp Fiber and Placebo Study Groups.

## References

[1] Ribeiro F.M., Petriz B., Marques G., Kamilla L.H., Franco O.L. (2021). Is there an exercise-intensity threshold capable of avoiding the leaky gut?. Front. Nutr..

[2] Keirns B.H., Koemel N.A., Sciarrillo C.M., Anderson K.L., Emerson S.R. (2020). Exercise and intestinal permeability: Another form of exercise-induced hormesis?. Am. J. Physiol. Gastrointest. Liver Physiol..

[3] Chantler S., Griffiths A., Matu J., Davison G., Holliday A., Jones B. (2022). A systematic review: Role of dietary supplements on markers of exercise-associated gut damage and permeability. PLoS ONE.

[4] Dziewiecka H., Buttar H.S., Kasperska A., Ostapiuk-Karolczuk J., Domagalska M., Cichoń J., Skarpańska-Stejnborn A. (2022). A systematic review of the influence of bovine colostrum supplementation on leaky gut syndrome in athletes: Diagnostic biomarkers and future directions. Nutrients.

[5] Tataka Y., Haramura M., Hamada Y., Ono M., Toyoda S., Yamada T., Hiratsu A., Suzuki K., Miyashita M. (2022). Effects of oral cystine and glutamine on exercise-induced changes in gastrointestinal permeability and damage markers in young men. Eur. J. Nutr..

[6] Nieman D.C., Gillitt N.D., Chen G.Y., Zhang Q., Sha W., Kay C.D., Chandra P., Kay K.L., Lila M.A. (2020). Blueberry and/or banana consumption mitigate arachidonic, cytochrome p450 oxylipin generation during recovery from 75-Km cycling: A randomized trial. Front. Nutr..

[7] Nieman D.C., Kay C.D., Rathore A.S., Grace M.H., Strauch R.C., Stephan E.H., Sakaguchi C.A., Lila M.A. (2018). Increased plasma levels of gut-derived phenolics linked to walking and running following two weeks of flavonoid supplementation. Nutrients.

[8] Nieman D.C., Gillitt N.D., Knab A.M., Shanely R.A., Pappan K.L., Jin F., Lila M.A. (2013). Influence of a polyphenol-enriched protein powder on exercise-induced inflammation and oxidative stress in athletes: A randomized trial using a metabolomics approach. PLoS ONE.

[9] Flores Martinez K.E., Bloszies C.S., Bolino M.J., Henrick B.M., Frese S.A. (2024). Hemp hull fiber and two constituent compounds, N-trans-caffeoyltyramine and N-trans-feruloyltyramine, shape the human gut microbiome in vitro. Food Chem. X.

[10] van Klinken B.J., Stewart M.L., Kalgaonkar S., Chae L. (2024). Health-promoting opportunities of hemp hull: The potential of bioactive compounds. J. Diet. Suppl..

[11] Bolster D., Chae L., van Klinken J.W., Kalgaonkar S. (2022). Impact of selected novel plant bioactives on improvement of impaired gut barrier function using human primary cell intestinal epithelium. J. Food Bioact..

[12] Lee S.H., Veeriah V., Levine F. (2022). A potent HNF4α agonist reveals that HNF4α controls genes important in inflammatory bowel disease and Paneth cells. PLoS ONE.

[13] Smith L.L., Brunetz M.H., Chenier T.C., McCammon M.R., Houmard J.A., Franklin M.E., Israel R.G. (1993). The effects of static and ballistic stretching on delayed onset muscle soreness and creatine kinase. Res. Q. Exerc. Sport..

[14] Curran S.L., Andrykowski M.A., Studts J.L. (1995). Short form of the Profile of Mood States (POMS-SF): Psychometric information. Psychol. Assess..

[15] Khoshbin K., Khanna L., Maselli D., Atieh J., Breen-Lyles M., Arndt K., Rhoten D., Dyer R.B., Singh R.J., Nayar S. (2021). Development and validation of test for “leaky gut” small intestinal and colonic permeability using sugars in healthy adults. Gastroenterology.

[16] Larkey N.E., Fatica E.M., Singh R.J. (2022). Detection of 13C-mannitol and other saccharides using tandem mass spectrometry for evaluation of intestinal permeability or leaky gut. Methods Mol. Biol..

[17] Pathmasiri W., Rushing B.R., McRitchie S., Choudhari M., Du X., Smirnov A., Pelleigrini M., Thompson M.J., Sakaguchi C.A., Nieman D.C. (2024). Untargeted metabolomics reveal signatures of a healthy lifestyle. Sci. Rep..

[18] Li Y.Y., Ghanbari R., Pathmasiri W., McRitchie S., Poustchi H., Shayanrad A., Roshandel G., Etemadi A., Pollock J.D., Malekzadeh R. (2020). Untargeted metabolomics: Biochemical perturbations in golestan cohort study opium users inform intervention strategies. Front. Nutr..

[19] Ghanbari R., Li Y., Pathmasiri W., McRitchie S., Etemadi A., Pollock J.D., Poustchi H., Rahimi-Movaghar A., Amin-Esmaeili M., Roshandel G. (2021). Metabolomics reveals biomarkers of opioid use disorder. Transl. Psychiatry.

[20] Lynch D.H., Rushing B.R., Pathmasiri W., McRitchie S., Batchek D.J., Petersen C.L., Gross D.C., Sumner S.C.J., Batsis J.A. (2023). Baseline serum biomarkers predict response to a weight loss intervention in older adults with obesity: A pilot study. Metabolites.

[21] Sun J., Xia Y. (2023). Pretreating and normalizing metabolomics data for statistical analysis. Genes Dis..

[22] Välikangas T., Suomi T., Elo L.L. (2018). A systematic evaluation of normalization methods in quantitative label-free proteomics. Brief. Bioinform..

[23] Smirnov A., Liao Y., Fahy E., Subramaniam S., Du X. (2021). ADAP-KDB: A spectral knowledgebase for tracking and prioritizing unknown GC-MS spectra in the NIH’s Metabolomics Data Repository. Anal. Chem..

[24] Chen X., Zhu X., Wu G., Wang X., Zhang Y., Jiang N. (2024). Structure-based identification of HNF4α agonists: Rosmarinic acid as a promising candidate for NAFLD treatment. Comput. Struct. Biotechnol. J..

[25] Deehan E.C., Mocanu V., Madsen K.L. (2024). Effects of dietary fibre on metabolic health and obesity. Nat. Rev. Gastroenterol. Hepatol..

[26] Mocanu V., Madsen K.L. (2024). Dietary fibre and metabolic health: A clinical primer. Clin. Transl. Med..

[27] Wang Z., Peters B.A., Yu B., Grove M.L., Wang T., Xue X., Thyagarajan B., Daviglus M.L., Boerwinkle E., Hu G. (2024). Gut microbiota and blood metabolites related to fiber intake and type 2 diabetes. Circ. Res..

[28] Myhrstad M.C.W., Tunsjø H., Charnock C., Telle-Hansen V.H. (2020). Dietary fiber, gut microbiota, and metabolic regulation-current status in human randomized trials. Nutrients.

[29] Mahalak K.K., Liu L., Bobokalonov J., Narrowe A.B., Firrman J., Bittinger K., Hu W., Jones S.M., Moustafa A.M. (2024). Supplementation with soluble or insoluble rice-bran fibers increases short-chain fatty acid producing bacteria in the gut microbiota in vitro. Front. Nutr..

[30] Vinelli V., Biscotti P., Martini D., Del Bo’ C., Marino M., Meroño T., Nikoloudaki O., Calabrese F.M., Turroni S., Taverniti V. (2022). Effects of dietary fibers on short-chain fatty acids and gut microbiota composition in healthy adults: A systematic review. Nutrients.

[31] Fernández M.A., García M.D., Sáenz M.T. (1996). Antibacterial activity of the phenolic acids fractions of Scrophularia frutescens and Scrophularia sambucifolia. J. Ethnopharmacol..

[32] Meng D., Sommella E., Salviati E., Campiglia P., Ganguli K., Djebali K., Zhu W., Walker W.A. (2020). Indole-3-lactic acid, a metabolite of tryptophan, secreted by Bifidobacterium longum subspecies infantis is anti-inflammatory in the immature intestine. Pediatr. Res..

[33] Alonso-Esteban J.I., Pinela J., Ćirić A., Calhelha R.C., Soković M., Ferreira I.C.F.R., Barros L., Torija-Isasa E., Sánchez-Mata M.C. (2022). Chemical composition and biological activities of whole and dehulled hemp (*Cannabis sativa* L.) seeds. Food Chem..

[34] van Wijck K., Lenaerts K., van Loon L.J., Peters W.H., Buurman W.A., Dejong C.H. (2011). Exercise-induced splanchnic hypoperfusion results in gut dysfunction in healthy men. PLoS ONE.

[35] Groschwitz K.R., Hogan S.P. (2009). Intestinal barrier function: Molecular regulation and disease pathogenesis. J. Allergy Clin. Immunol..

[36] Shu L.Z., Ding Y.D., Xue Q.M., Cai W., Deng H. (2023). Direct and indirect effects of pathogenic bacteria on the integrity of intestinal barrier. Ther. Adv. Gastroenterol..

[37] Peters H.P., Bos M., Seebregts L., Akkermans L.M., van Berge Henegouwen G.P., Bol E., Mosterd W.L., de Vries W.R. (1999). Gastrointestinal symptoms in long-distance runners, cyclists, and triathletes: Prevalence, medication, and etiology. Am. J. Gastroenterol..

[38] Nieman D.C., Henson D.A., Dumke C.L., Oley K., McAnulty S.R., Davis J.M., Murphy E.A., Utter A.C., Lind R.H., McAnulty L.S. (2006). Ibuprofen use, endotoxemia, inflammation, and plasma cytokines during ultramarathon competition. Brain Behav. Immun..

[39] Chelakkot C., Ghim J., Ryu S.H. (2018). Mechanisms regulating intestinal barrier integrity and its pathological implications. Exp. Mol. Med..

[40] Quaresma M.V.L.D.S., Mancin L., Paoli A., Mota J.F. (2024). The interplay between gut microbiome and physical exercise in athletes. Curr. Opin. Clin. Nutr. Metab. Care.

[41] Nolte S., Krüger K., Lenz C., Zentgraf K. (2023). Optimizing the Gut Microbiota for Individualized Performance Development in Elite Athletes. Biology.

[42] Han M., Yang K., Yang P., Zhong C., Chen C., Wang S., Lu Q., Ning K. (2020). Stratification of athletes’ gut microbiota: The multifaceted hubs associated with dietary factors, physical characteristics and performance. Gut Microbes.

[43] Plamada D., Vodnar D.C. (2021). Polyphenols-gut microbiota interrelationship: A transition to a new generation of prebiotics. Nutrients.

